# Novel IKZF3 transcriptomic signature correlates with positive outcomes of skin cutaneous melanoma: A pan-cancer analysis

**DOI:** 10.3389/fgene.2022.1036402

**Published:** 2022-10-24

**Authors:** Lin-Kai Yang, Can-Xiang Lin, Sheng-Hong Li, Jia-Ji Liang, Li-Ling Xiao, Guang-Hui Xie, Hong-Wei Liu, Xuan Liao

**Affiliations:** Department of Plastic Surgery, The First Affiliated Hospital of Jinan University, Innovative Technology Research Institute of Tissue Repair and Registration, Key Laboratory of Regenerative Medicine, Ministry of Education, Guangzhou, Guangdong Province, China

**Keywords:** ikaros family genes, SKCM, immune infiltration, immunotherapy biomarker, pan-cancer analysis

## Abstract

To investigate the potential relationship between Ikaros family genes and skin cutaneous melanoma (SKCM), we undertook a pan-cancer analysis of the transcriptional signature and clinical data of melanoma through multiple databases. First, 10,327 transcriptomic samples from different cancers were included to determine the overall characteristics and clinical prognoses associated with Ikaros gene expression across cancer types. Second, differentially expressed genes analysis, prognostic evaluation, and gene set enrichment analysis were employed to investigate the role of Ikaros (*IKZF*) genes in SKCM. Third, we evaluated the relationship between Ikaros family genes and SKCM immune infiltrates and verified the findings using the GEO single-cell sequencing dataset. The results show that Ikaros genes were widely expressed among different cancer types with independently similar patterns as follows: 1. *IKZF1* and *IKZF3*, and 2. *IKZF2* and *IKZF4–5*. *IKZF2* and *IKZF5* were downregulated in the primary tumor, and *IKZF1–3* expression decreased significantly as the T-stage or metastasis increased in SKCM. Moreover, high *IKZF1–3* expression was associated with better overall survival, disease-specific survival, and progression-free interval. *IKZF3* is an independent prognostic factor of SKCM. Among Ikaros genes, the expression of *IKZF1* and *IKZF3* positively correlated with the infiltration level of CD4^+^ T cells and CD8^+^ T cells, B cells, and Tregs in SKCM and negatively correlated with the infiltration level of M0 and M1 macrophages. Moreover, single-cell sequencing data analysis revealed that *IKZF1* and *IKZF3* were mainly expressed by immune cells. Correlation analysis shows the immune factors and drug responses associated with IKZF3 expression. In conclusion, the present study is the first, to our knowledge, to identify a pan-cancer genomic signature of the Ikaros gene family among different cancers. Expression of these family members, particularly high levels of *IKZF3*, indicate positive immunological status and beneficial clinical outcomes of SKCM. *IKZF3* may therefore serve as potential targets for immunotherapy of melanoma.

## Introduction

As one of the most serious cancers, SKCM easily metastasizes to other parts of body through the lymphatic and peripheral circulatory systems. In recent years, SKCM morbidity tends to affect more of the younger population and has become one of the tumors with the fastest increase in incidence across cancer types (Ferlay et al., 2015; Siegel et al., 2016). Early-stage patients with SKCM do not exhibit apparent symptoms or classical characteristics in pathological examinations, which hinders physicians from diagnosing and treating patients in a timely manner (Megahed et al., 2002).

For example, conventional surgical resection of local lesions and radiation therapy hardly achieve significantly positive outcomes of SKCM, particularly for multiorgan metastasis (Balch et al., 2001). In contrast, the antiapoptotic activity of SKCM renders it resistant to most chemotherapies and increases the risk of its recurrence (Soengas and Lowe, 2003; Nazarian et al., 2010). Although SKCM is currently treated with a combination of surgery, radiation, and other systematic modalities, the survival rate of patients with advanced melanoma metastasis remains extremely low.

However, with constant improvements in immunomodulatory technology, cancer immunotherapy appears to serve as an effective method to treat malignant tumors. Mounting studies highlight the role of tumor-infiltrating lymphocytes in eliminating tumors from the tumor microenvironment (TME), thus making it reasonable to treat SKCM with an immunotherapy strategy.

In tumor tissues, fibroblasts, infiltrating lymphocytes, and components such as the surrounding stromal cells and capillaries constitute the TME (Locy et al., 2018). As the “soil” for tumor cell growth, the TME mainly provides nutrients for tumor cell proliferation and invasion. However, once the dysfunctional infiltrated immune cells recover, the tumor microenvironment may become a battlefield to kill tumors. In recent years, mounting studies reveal that quiescent tumor-infiltrating lymphocytes (TILs) are the main forces that kill tumor cells after they respond to effective immunological stimulation *via* the TME (Lee et al., 2016; Fu et al., 2019). In SKCM, effector T cells, a member of the TIL population, continuously eradicate tumor cells once activated by antigens presented by other cells, thus improving the prognostic outcome of patients (Zhou et al., 2005).

Methods for transforming the TME from a quiescent to active immunological state range from intravenous injection of recombinant interleukin (Atkins et al., 2000) and adoptive T-cell therapy (Besser et al., 2010) to monoclonal antibody therapy (Weber, 2010). By blocking the PD-1/PD-L1-induced signaling pathway or activating the costimulatory receptor 4-1BB/4-1BBL, monoclonal antibody therapy of tumors may improve clinical outcomes. However, most patients with SKCM do not positively respond to single-target therapy, particularly those with metastasized melanoma (Tsai et al., 2014). Therefore, it is vitally importance to investigate effective immunological therapies that target SKCM.

The Ikaros family of zinc-finger proteins plays a critical role in lymphatic development, differentiation, and homeostasis. There are five homologous members of the Ikaros gene family including Ikaros (*IKZF1*), Helios (*IKZF2*), Aiolos (*IKZF3*), Eos (*IKZF4*), and Pegasus (*IKZF5*), which are translated as distinct isoforms through alternative splicing (John and Ward, 2011). The Ikaros zinc finger-like N-terminal domain specifically binds DNA, and its zinc finger-like C-terminal domain mediates homologous binding as well as to other family members (Molnár et al., 1994; Sun et al., 1996). These features confer upon Ikaros family members the ability to regulate cell proliferation, differentiation, and apoptosis through the combination of Ikaros family members or other transcription factors (Yoshida and Georgopoulos, 2014).

*IKZF4* and *IKZF5* are mainly expressed in skeletal muscle and other solid organs, and *IKZF1–3* expressed by lymphocytes participate in the regulation of the development and differentiation of these cells (Perdomo et al., 2000; Yoshida and Georgopoulos, 2014). It is therefore reasonable to conclude that Ikaros genes influence the immunological status in the TME to affect patients’ clinical outcomes. However, the potential relationship between TILs that express Ikaros genes in the TME and SKCM has not been comprehensively investigated.

In the present study, we first applied bioinformatics to analyze RNA-seq data for different cancers to determine the role of Ikaros genes in their pathogenesis. The potential function of Ikaros genes in SKCM was confirmed using gene set enrichment analysis (GSEA), survival evaluation, and other analytical tools. To determine the effects of Ikaros gene expression on the TIL population of SKCM, we performed immunological analyses using CIBERSORT and single-cell RNA-seq. Finally, a novel *IKZF1–3* transcriptomic signature was shown to correlate with positive outcomes of SKCM.

## Methods

### Data analysis

RNA sequence data and corresponding clinical data of patients with different carcinomas (33 types) were acquired from the UCSC Xena database (https://xenabrowser.net/datapages/). The Ensemble IDs of the expression profile data were converted to symbol IDs through the human GTF file. Cancer types were included: ACC, BLCA, BRCA, COAD, DLBC, ESCA, GBM, HNSC, KICH, KIRC, KIRP, LAML, LGG, LIHC, LUAD, LUSC, OV, PAAD, PRAD, READ, SKCM, STAD, TGCT, THCA, THYM, UCEC, and UCS. At the same time, clinical data were included survival time, progression free survival time, survival status, clinical staging and other clinical follow-up data.

### Ikaros transcriptional signature across cancer types

We determined the expression levels of *Ikaros* family members in all cancers and identified the individual expression characteristics of Ikaros family members through Pearson coefficient correlation analysis. In view of the lack of normal samples of SKCM in TCGA data and to evaluate the association between Ikaros and the progression of SKCM, differential expressional levels of normal and tumor samples were retrieved and compared using the GEPIA database (http://gepia.cancer-pku.cn). Furthermore, we selected cancer types with more than three normal data samples and analyzed the differences between the expression of Ikaros family genes in cancer and normal tissues. |logFC>2| and *p* < 0.05 were considered to indicate significant differences in expression.

### Prognostic analysis

The prognostic outcomes of Ikaros expression among multiple cancers were determined using Cox univariate analysis with the indexes of hazard ratios (HRs) and 95% confidence intervals (CIs). The log-rank test and the Kaplan–Meier algorithm were used to determine the correlation between expression of individual Ikaros genes and a patient’s survival. Several indicators of outcomes, such as overall survival (OS), progression-free interval (PFI), and disease-free survival (DSS) were applied to comprehensively evaluate the prognostic significance of Ikaros gene expression.

### GSEA and protein-protein interaction network analysis

A computational method of GSEA (GSEA v.3.0 in the Java environment) was used to explore the potential mechanism of the regulation of Ikaros genes in the occurrence and development of SKCM. We ranked the levels of Ikaros genes expressed by SKCM samples from high to low for GSEA, which was used to identify signaling pathways correlated with Ikaros. Gene-set permutations were performed 1,000 times for each analysis. The expression levels of *IKZF1–IKZF5* were used as phenotypic labels for all SKCM samples. A false discovery rate (FDR) < 0.25 and *p* < 0.05 were used to identify KEGG pathways significantly enriched in each phenotype. Ikaros-interacting proteins were determined using the GeneMANIA protein-protein interaction network (http://genemania.org/).

### Analysis of immune infiltration

An algorithm of ESTIMATE (Yoshihara et al., 2013) was adopted to evaluate immune infiltration condition across cancer types using the indexes of stromal score, immune score, and tumor purity. The relationship between Ikaros gene expression and immunological reactivity was determined by comparing the scores and purity indexes among different carcinomas. CIBERSORT was used for characterizing the infiltrating immune cell composition of SKCM tissue from their gene expression profiles. We performed deconvolution calculations with the annotating file LM22. CIBERSORT annotates the abundance of 22 infiltrating immune cells through a 547-gene expression eigenmatrix (Newman et al., 2015). Through the results of CIBERSORT, the correlations between immune-infiltrating cells in the TME of SKCM and the expression of Ikaros genes were demonstrated.

### Single-cell RNA-seq analysis

The Tumor Immune-single Cell Hub (TISCH) is an SCRNA-Seq database focused on the TME, which provides detailed cellular annotations at the level of a single cell that enables exploration of the TMEs of different cancer types (Sun et al., 2021). GSE72056, a series of 4,645 single-cell sRNA-seq data from 19 melanoma samples included in the Gene Expression Omnibus by Tirosh et al. was further analyzed using TISCH to determine Ikaros gene expression levels in immunocytes.

### Correlation of *IKZF3* expression and immune factors

The TISIDB database (http://cis.hku.hk/TISIDB) is a web portal for tumour and immune system interaction, which integrates multiple heterogeneous data types. Gene signatures of Tumor-infiltrating lymphocytes (TILs) were obtained from TISIDB database. Here, we further analyzed associations for IKZF3 with immunomodulators, chemokines and receptors. Immunomodulators can be divided into three categories, including immunoinhibitors, immunostimulators and major histocompatibility complex (MHC) molecules.

### Correlation of *IKZF3* expression and drug responses

The cellminer database contains (NCI)-60 gene expression and drug sensitivity data. We obtained the cellminer database including drug data approved by the US Food and Drug Administration and drug data evaluated in clinical trials. The Pearson correlation coefficient was used to evaluate the correlation between *IKZF3* expression and drug response.

### Statistical analysis

The Wilcoxon rank-sum test and the Kruskal–Wallis test were used to compare two and multiple groups, respectively. Pearson analysis was used to perform correlation analysis among members of the Ikaros gene family. *p* < 0.05 indicates a significant difference. All statistical analyses were performed using R (version 3.6.0).

## Results

### Transcriptomic signatures of ikaros genes across cancer types

**FIGURE 1:**
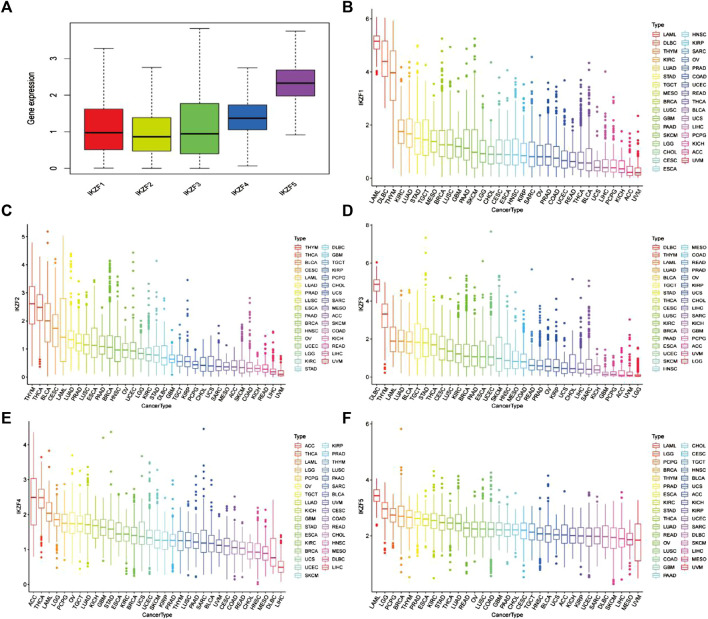
**(A)** Mean mRNA expression values of Ikaros in pan-cancers. **(B–F)** mRNA expression levels of *IKZF1–5* in different human cancers ranked from high to low.

Differential transcriptomic levels of Ikaros members (*IKZF1–5*) were determined through the analysis of 11,057 samples of 33 tumors (10,327 tumor and 730 normal samples). To determine the expression characteristics of Ikaros members, we first calculated the mean expression levels of *IKZF1–5* in pan-cancers. Among them, the mean expression levels of *IKZF1–3* were relatively consistent and that of *IKZF5* was the highest (Figure 1A). Furthermore, the transcriptional signatures of individual Ikaros genes in different cancer types revealed that *IKZF1–5* were mainly overexpressed in hematological tumors such as LAML and DLBC, but were expressed at low levels in digestive tumors, for example, LIHC. The detailed expression levels of Ikaros genes in different cancer types are shown in Figures 1B–F.

**FIGURE 2:**
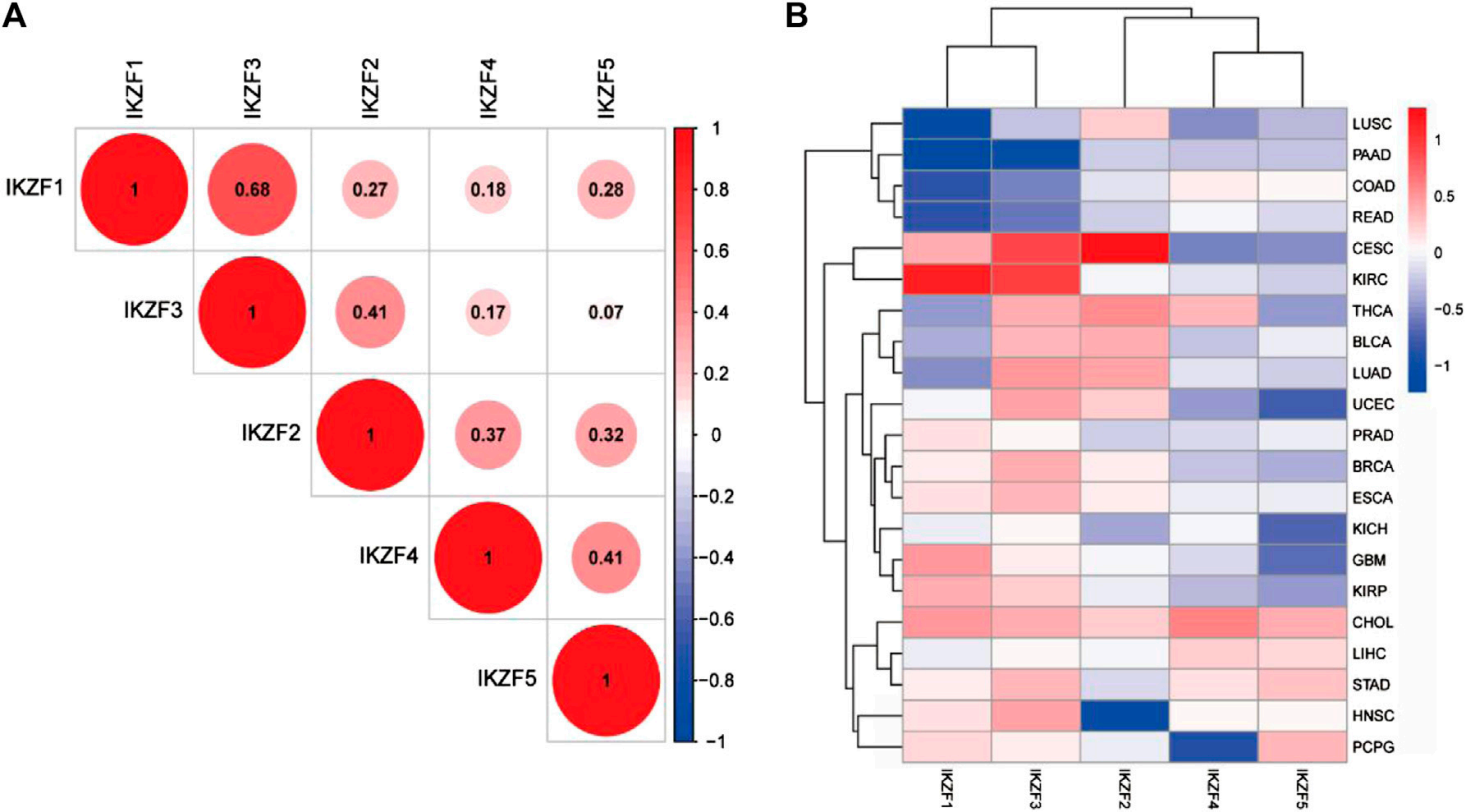
**(A)** Pearson coefficient indexes between Ikaros genes. **(B)** Heatmap of differentially expressed Ikaros genes in 21 TGCA datasets. Red and blue indicate upregulation and downregulation, respectively.

The Pearson coefficient index of every pair of Ikaros genes indicated that *IKZF1* and *IKZF3* were closely associated in various cancers, while *IKZF3* and *IKZF5* showed the weakest correlations (Figure 2A). We next screened 21 tumor datasets, each including at least three normal samples, using hierarchical cluster analysis of differentially expressed genes. The results confirmed the transcriptional relationship between *IKZF1* and *IKZF3*, because they ranked in the same cluster with a transcriptional pattern similar to that of *IKZF1* and *IKZF3* in different cancer types (Figure 2B).

**FIGURE 3:**
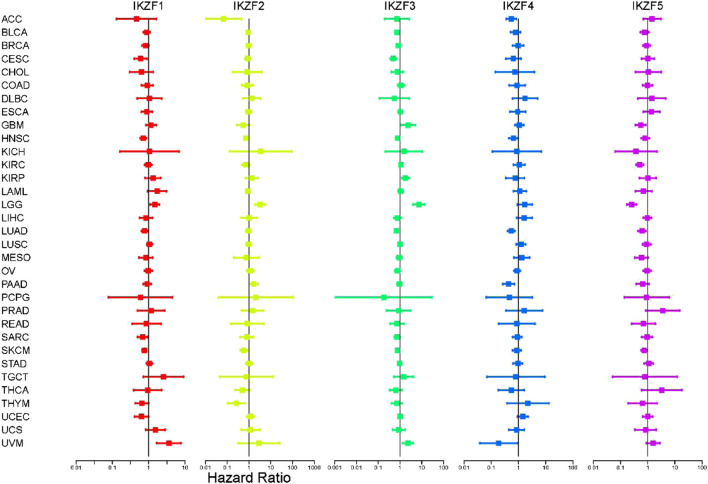
Cox univariate regression analysis of the overall survival associated with Ikaros expressed in different cancers. The vertical axis indicates the cancer type. Different colors represent different Ikaros members, and the dots represent the risk ratio (HR) of the gene in cancer. The two lines End represents the 95% confidence interval (CI).

To further investigate the role of *IKZF1–5* in predicting clinical outcomes, we conducted univariate Cox regression analysis of gene samples with the corresponding clinical data. We found that most patients with cancer that highly expressed *IKZF1–3* had a significantly improved prognosis. For example, high levels of *IKZF1–3* in patients with CESC, HNSC, LUAD, SARC, and SKCM indicated longer survival but implied poor prognosis of patients with LGG and UVM with the same transcriptional profile (Figure 3). Although there was no significant association between *IKZF4* and *IKZF5*, their high expression levels indicate better outcomes of certain types of cancers. the high expression of *IKZF1, IKZF2, IKZF3,* and *IKZF5* in SKCM will indicate a better prognosis.

### *IKZF1–3* expression negatively correlates with the progression of SKCM

**FIGURE 4:**
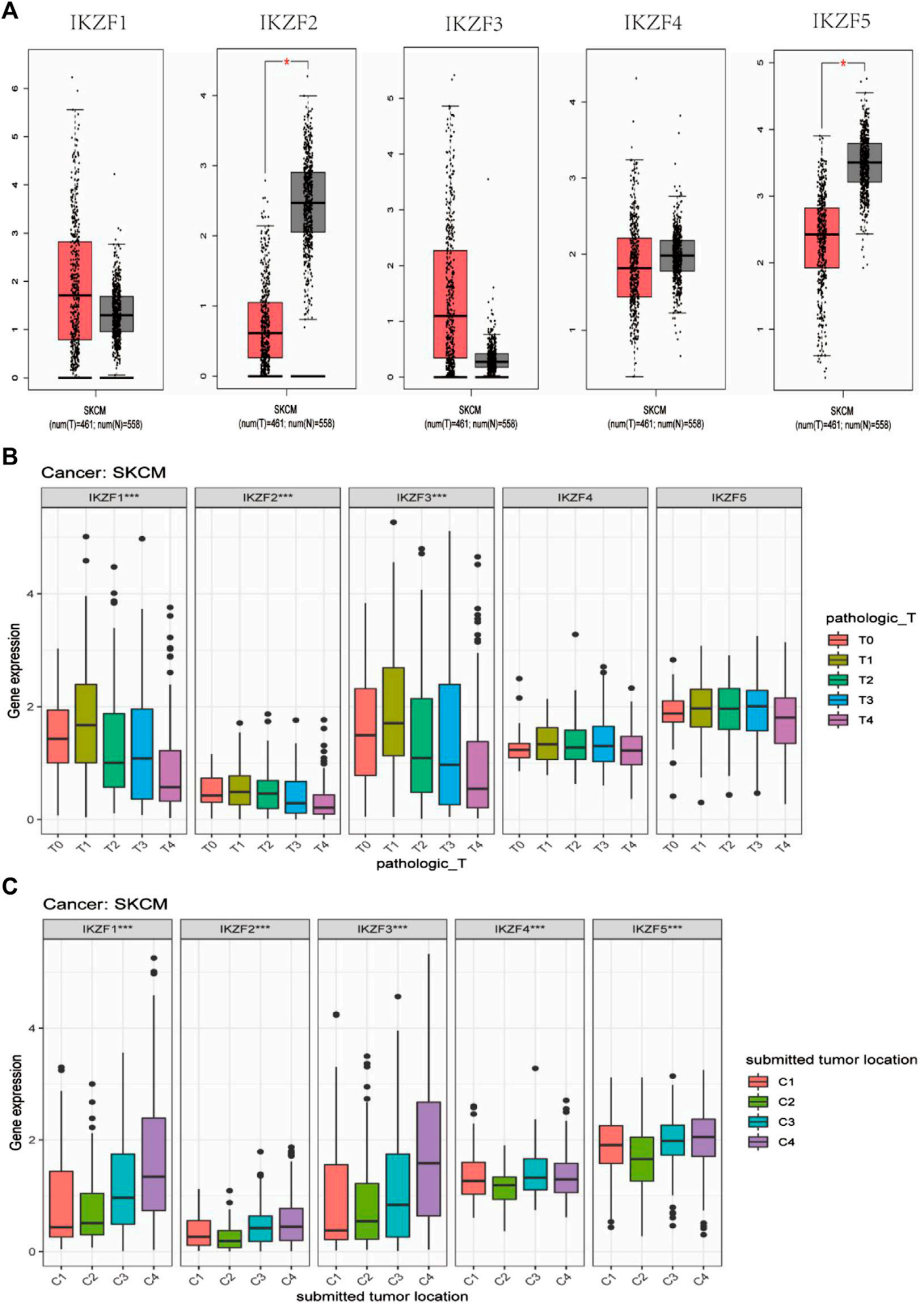
**(A)** GEPIA box diagram shows the expression levels of Ikaros genes in SKCM and healthy skin. T: tumor tissue, N: normal tissue; **(B)** Ikaros gene expression in patients with different pathological T-stages; **(C)** Ikaros gene expression at different tumor locations, C1 (Distant Metastasis), C2 (Primary Tumor), C3 (Regional Cutaneous or Subcutaneous Tissue [includes satellite and in-transit metastasis]), C4 (Regional Lymph Node) (*p < 0.05, **p < 0.01, ***p < 0.001).

Compared with normal tissue, *IKZF2* and *IKZF5* mRNAs were expressed at lower levels in SKCM (Figure 4A). Considering that Ikaros genes may participate in the progression of SKCM, we investigated their transcriptional levels associated with different TNM stages. The results show that the expression of *IKZF1–3* mRNAs in SKCM significantly decreased with higher T-stage and significantly decreased when SKCM involved local lymph node metastasis that penetrated the original tissue boundary (Figure 4B). However, the levels of mRNAs of other members of the Ikaros family did not vary with higher T-stage. Moreover, among different tumor locations, Ikaros genes were expressed at the highest levels in regional lymph nodes, *IKZF1* and *IKZF3* were expressed at the lowest levels in distant metastases, and *IKZF2*, *IKZF4*, and *IKZF5* were expressed at the lowest levels in the primary tumor.

### Highly expressed *IKZF1–3* are associated with favorable clinical outcomes of patients with SKCM

**FIGURE 5:**
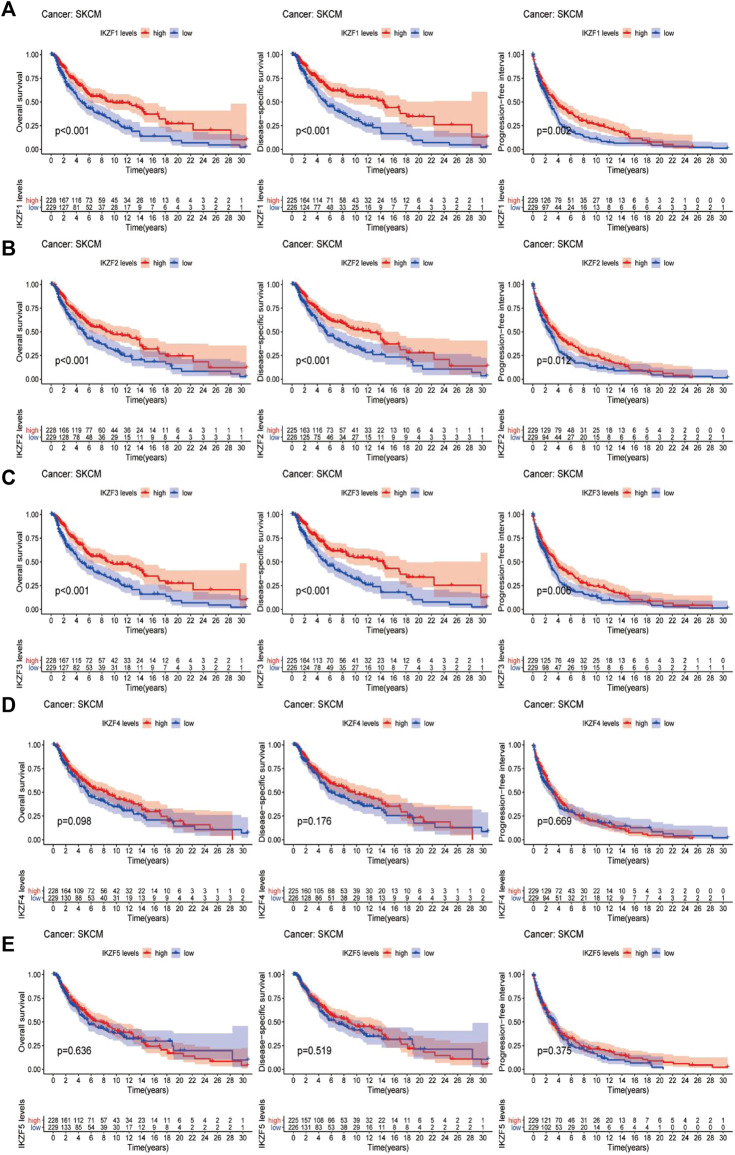
Comparison of Kaplan–Meier survival curves of patients with high or low expression of Ikaros. OS, DSS, and PFI rates associated with the expression of Ikaros genes in the SKCM cohort. **(A–E)**, *IKZF1–5*, respectively.

The results of Kaplan-Meier analysis suggested that patients with SKCM with highly expressed *IKZF1–3* experienced significantly longer OS, DFS, and PFI than patients with lower levels (*p* < 0.05) (Figures 5A–C). However, the associations of the OS, DSS, and PFI rates of patients with higher expression levels of *IKZF4–5* were not significantly different (Figures 5D,E), and patients who expressed high levels of *IKZF1–3* experienced significantly longer DSS and longer tumor progression-free survival. These findings indicate that high expression of *IKZF1–3* mRNAs was significantly associated with good prognosis of patients with SKCM. Further multivariate-cox analysis indicates that IKZF3 can be used as an independent prognostic factor (Supplementary Figure S1).

### High expression of *IKZF1–3* activates an antitumor immune response

**FIGURE 6:**
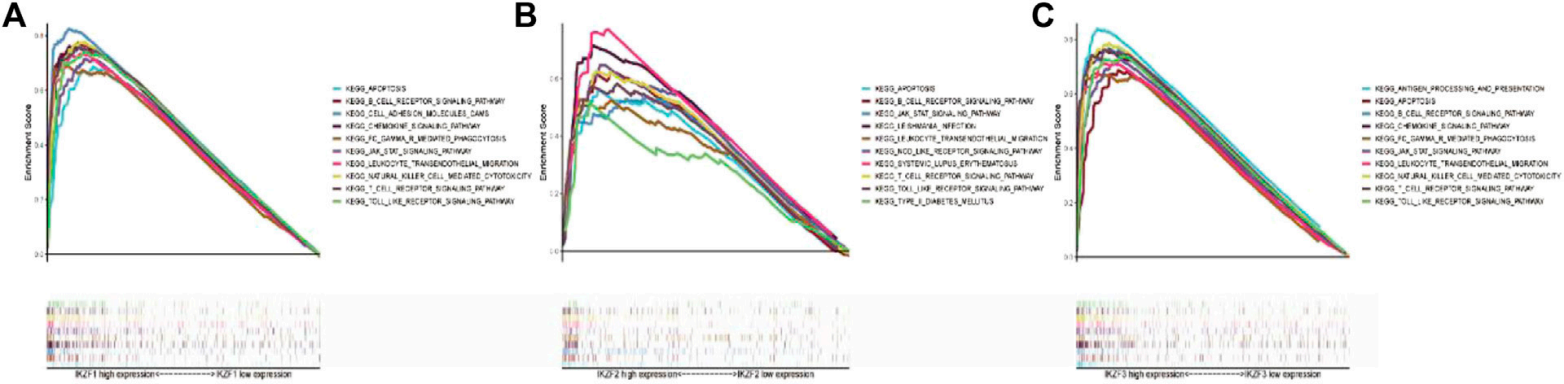
GSEA results of single Ikaros genes. **(A–C)**: The first 10 KEGG pathways clustered depending on the relationships of the transcriptional changes of *IKZF1–3*.

To further investigate Ikaros-associated signaling pathways, transcriptional data of SKCM samples were ranked by the relative levels of *IKZF1–5* expression in the upper 10th percentile (IKZFhi) vs. the lower 10th percentile (IKZFlo) and subjected to GSEA. Figures 6A–C shows that high expression of *IKZF1–3* was associated with several activated KEGG pathways, while *IKZF4* and *IKZF5* were not clustered in known pathways (data not shown). Moreover, the GSEA results demonstrate that high expression of *IKZF1–3* plays a critical role in the regulation of the activation of the immune response through the B-cell receptor signaling pathway, the T-cell receptor signaling pathway, leukocyte transendothelial migration, and natural killer cell-mediated cytotoxicity. Furthermore, IKZF1-3hi was closely associated with apoptosis and chemokine signaling pathways in the SKCM cohort, which indicates the anticancer effect of *IKZF1–3* through its regulation of the immune response.

### Increased *IKZF1–3* expression enhances immunological infiltration of SKCM

Tumor infiltration analysis using CIBESORT and Estimate, shows that the stromal and immune scores significantly increased as the expression of *IKZF1–3* was enhanced in SKCM samples. Moreover, there was a positive correlation between increased *IKZF1–3* expression and the abundance of immunocytes such as B cells, CD4-positive T cells and CD8-positive T cells, although there was a negative correlation between increased *IKZF1–3* expression and tumor infiltration with M0/M2-polarized macrophages. Furthermore, increased infiltration of M0/M2 macrophages was a poor prognostic factor for patients. (Supplementary Figure S2). However, there was no significant relationship between tumor infiltration and expression of *IKZF4–5*.

### Single-cell sequence analysis of ikaros expression

**FIGURE 7:**
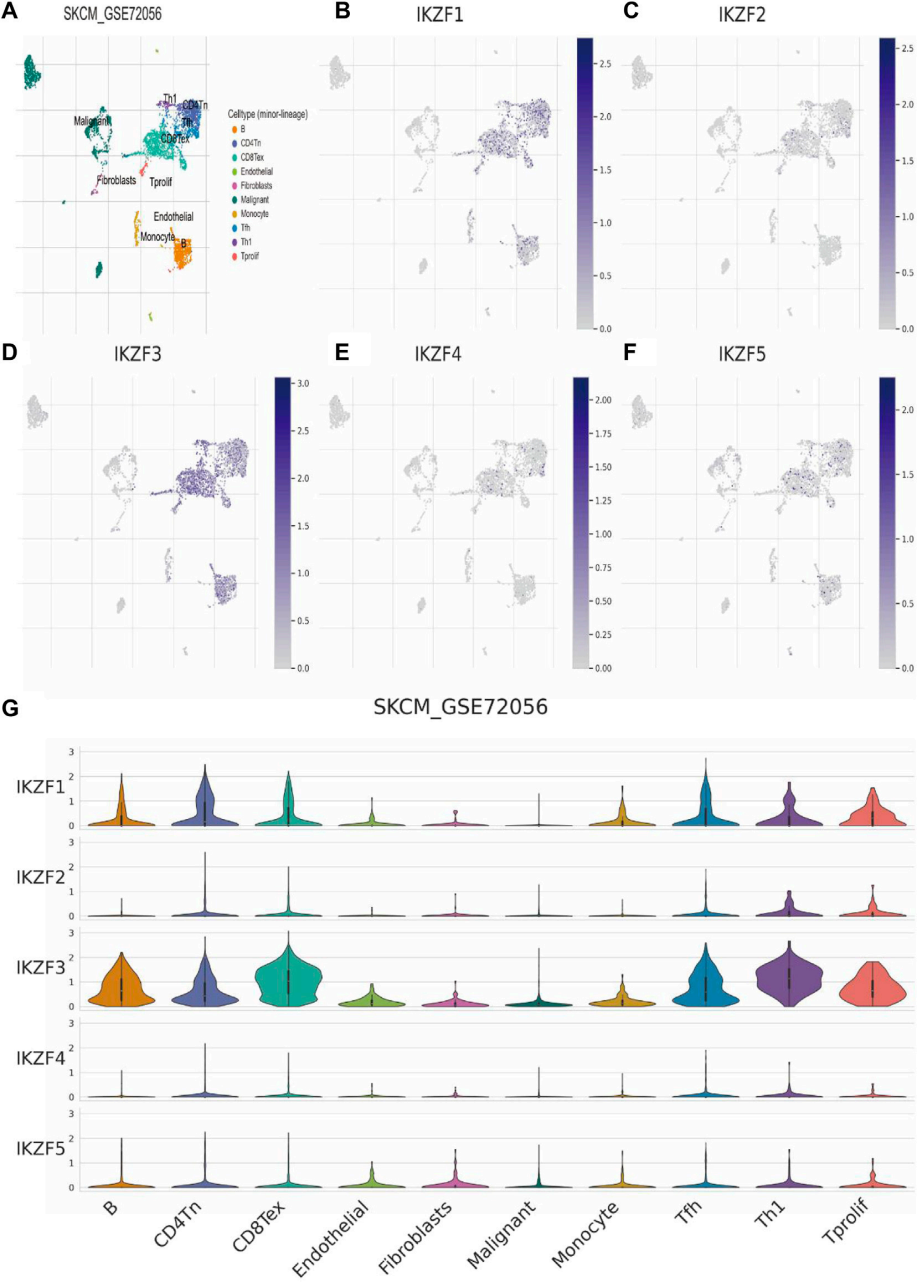
Transcriptomic signatures of different cell types in the melanoma microenvironment. **(A)**: There were 10 major cell types in the SKCM microenvironment as follows: B cells, CD4-positive T cells, CD8-positive T cells, endothelial cells, fibroblasts, malignant melanoma cells, monocytes, follicular helper T cells, Th1 helper cells, and proliferating T cells. **(B–E)**: Expression levels of *IKZF1–5* among different cell types in the melanoma.

Single-cell sequence analysis was applied to specifically determine the transcriptomic signature of Ikaros members. For this purpose, we employed melanoma tissue of the GSE72056 dataset. Using the Louvain clustering algorithm and a KNN graph, melanoma tissue was divided into the major cell types as follows: B cells, CD4-positive T cells, CD8-positive exhausted T cells, endothelial cells, fibroblasts, SKCM cells, monocytes, follicular helper T cells, Th1 helper cells, and proliferating T cells (8A, G). Consistent with the results mentioned above, the levels of *IKZF1* and *IKZF3* were highest in immunocytes and lowest in SKCM cells, which further indicates the function of *IKZF1* and *IKZF3* in the immune response to tumor cells and indicates the status of immunocyte infiltration in the TME (Figures 7B,D). However, *IKZF2*, *IKZF4*, and *IKZF5* were basically not detectably expressed by immunocytes, malignant cells, and stromal cells (Figures 7C,E,F).

### Correlation of *IKZF3* expression and immune factors

**FIGURE 8:**
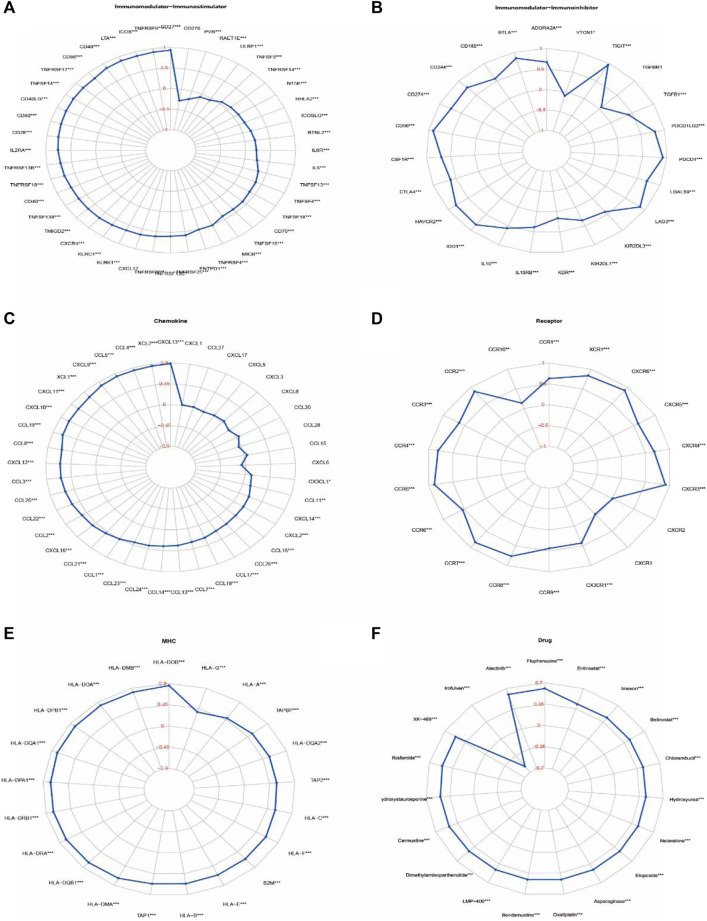
Correlations of IKZF3 expression with immunomodulatory genes, drugs. **(A–E)** Correlation between immunomodulators and chemokines (or receptors) with *IKZF3* expression. **(F)** Correlation between *IKZF3* expression and drug responses. The red numbers of radar chart represent Spearman’s correlation coefficient (*p < 0.05, **p < 0.01, ***p < 0.001).

Relationship of IKZF3 expression with immune factors was evaluated using TISIDB and TCGA databases. we calculated the correlation of IKZF3 expression and Gene signatures of TILs, including three immunomodulators, chemokines and receptors (Figures 8A–E). Figure 8A shows correlations between IKZF3 and immunostimulators, IKZF3 strongly correlated with CD27, TNFRSF9 and ICOS, and weakly correlated with TNFSF9 and ULBP1. Figure 8B shows correlations between IKZF3 and Immunoinhibitors, IKZF3 strongly correlated with CD96, PDCD1 and TIGIT, and weakly correlated with KDR and VTCN1. Figure 8C shows correlations between IKZF3 and chemokines, IKZF3 strongly correlated with CXCL13, XCL2 and CCL4, and weakly correlated with CXCL1 and CCL27. Figure 8D shows correlations between IKZF3 and receptors, IKZF3 strongly correlated with CXCR3, CCR5 and CCR4, and weakly correlated with CXCR1 and CXCR2. Figure 8E shows IKZF3 expression moderately to strongly correlated with MHC.

### Correlation of *IKZF3* expression and drug responses

In order to understand the potential relationship between IKZF3 expression and various types of drug response, we performed a correlation analysis to use CellMiner to identify potential drug candidates. IKZF3 expression in tumor cells was positively associated with increased drug sensitivity to Fluphenazine、Alectinib, and negatively associated with Irofulven. We have screened a total of 20 drugs that are most related to the expression of IKZF3, which are also shown in the radar chart (Figure 8F).

## Discussion

With an extremely low survival rate and high potential for metastatic growth, SKCM causes heavy economic losses and medical burdens annually worldwide. Except for conventional surgical removal of the carcinoma and regular radiation therapy, immunotherapy is one of the most promising treatments for SKCM (Besser et al., 2010; Hodi et al., 2010).

The recent research have determined the effect of immunotherapy in the treatment of tumors. As the development of cancer is highly associated with immune micro-environment, determination of the pivotal role of immune response in the tumor is of importance in developing new immunotherapeutic strategies (Roma-Rodrigues et al., 2019). IMiD (Immunomodulatory drugs) covers thalidomide, Len and pomalidomide, and is clinically approved for the treatment of MM (multiple myeloma) and other malignant tumors. Among them, MM and MDS (myelodysplastic syndrome) are widely used and studied. IMiD can achieve the therapeutic effect by inducing selective ubiquitination and proteasome degradation of Ikaros (Gao et al., 2020). In addition, IMiD has the ability to directly inhibit the growth of tumor cells and strong immune stimulation characteristics, so it has multiple effects on the existence of different cell components in the tumor microenvironment. The efficacy and safety of IMiD have been verified in a wide range of clinical trials. It is increasingly clear that IMiDs are promising in the treatment of MM (Gao et al., 2020), MDS and CLL (chronic lymphocytic leukemia) (Vitale et al., 2016). At the same time, Ikaros can be used as prognostic factors for MM patients treated by Len (Tachita et al., 2020). Previous Research has shown that patients with lower IKZF3 expression level have a poorer therapeutic effect with Len, which will also lead to shorter progression-free survival and overall survival (Pourabdollah et al., 2016). Moreover, inhibiting RUNX to protect Ikaros from degradation can significantly improve the drug resistance of IMiDs in MM(Zhou et al., 2019). In addition, the combined use of imatinib in children with B-ALL can enhance the therapeutic effect of IKZF1-deletion patients. Imatinib, as an intensive treatment for B-ALL in IKZF1- deletion children, significantly reduces the risk of recurrence and improves the 5-year overall survival of patients (Yeoh et al., 2018). CX4945, an inhibitor of CK2, can restore Ikaros function and play an anti-leukemia role *in vitro* or preclinical leukemia model (Borgo et al., 2021). In breast cancer, the application of ginseng polysaccharides can inhibit the proliferation of MDA-MB-231 cells by activating IKZF1 (Zhou et al., 2020). Through the inhibition of the CTLA4 *via* ipilimumab and the application of adoptive cellular immunotherapy with tumor-infiltrated lymphocytes, it enhanced the anti-tumor effects of T cells (Besser et al., 2010; Hodi et al., 2010).

The previous studies have revealed that SKCM patients with a high abundance of T and B cells or lowly infiltrated M0 and M2 macrophages have a significant prognosis, while the metastatic SKCM patients with low infiltration of B and CD8^+^ T cells have a worse outcome than the previous condition (Iglesia et al., 2016; Wang et al., 2020). The present study suggests that expressed *IKZF1-3* could activate the B cell receptor signaling pathway, T cell receptor signaling pathway, leukocyte trans-endothelial migration, antigen processing and presentation and several other immune regulation processes. Obviously, *IKZF1-3* is positively correlated with T and B cells infiltration, but negatively correlated with the M0 and M2 infiltration. Moreover, Ikaros is specifically expressed in the immunocytes. It seems that *IKZF1-3* participate the regulation of immunocytes’ differentiation, thus making it could be considered as an indicator for the status of TME in SKCM patients. Besides the regulation of hematopoietic process, Ikaros also regulates the balance of autoimmunity and suppresses the growth of tumor (Fan and Lu, 2016). Ikaros genes mediate lymphocyte proliferation and differentiation (Fan and Lu, 2016). *IKZF1-3* play an important role in the regulation of lymphatic system differentiation. The disorder of its expression has the directive relationship with the hematological malignancies and primary immunodeficiency. Lack of Ikaros family may lead to a variety of immune related diseases, including immune thrombocytopenia (Sriaroon et al., 2019), systemic lupus erythematosus (Chen et al., 2020), rheumatoid arthritis (Yang et al., 2019). For example, mutations of *IKZF1–3* in leukemias are associated with poor prognosis, mainly caused by the disruption of lymphocyte fates (Rebollo and Schmitt, 2003; Payne and Dovat, 2011). In addition, Some solid tumors are related to the abnormal expression of Ikaros family proteins. Higher levels of Ikaros have been proved to be related to poor differentiation and late stage of ovarian cancer (He et al., 2012). Moreover, hypermethylation of Ikaros levels can be regarded as a marker of colorectal cancer progression and poor prognosis (Javierre et al., 2011). As a nuclear transcription factor, IKZF1 could inhibit the proliferation of HCC through suppressing the promotor of ANXA4D (Liu et al., 2017). In lung cancer, overexpressed IKZF3 upregulates the expression of TWIST and matrix metalloproteinase-16, which promotes the epithelial-mesenchymal-transition and the transformation of cancer stem cells and leads to poor prognosis (Hung et al., 2020). Moreover, the highly expressed Ikaros are closely associated with recurrence and metastasis lung adenocarcinoma (Zhao et al., 2020). But, in several tumors that normally lack IKZF1 expression, overexpression of Ikaros leads to enhanced immune recruitment infiltration and tumor sensitivity to CTLA4 and PD1 inhibitors (Chen et al., 2018). However, no research, to our knowledge, has investigated the correlation between Ikaros expression and SKCM with the aim of developing markers to predict prognosis and tumor progression.

**FIGURE 9:**
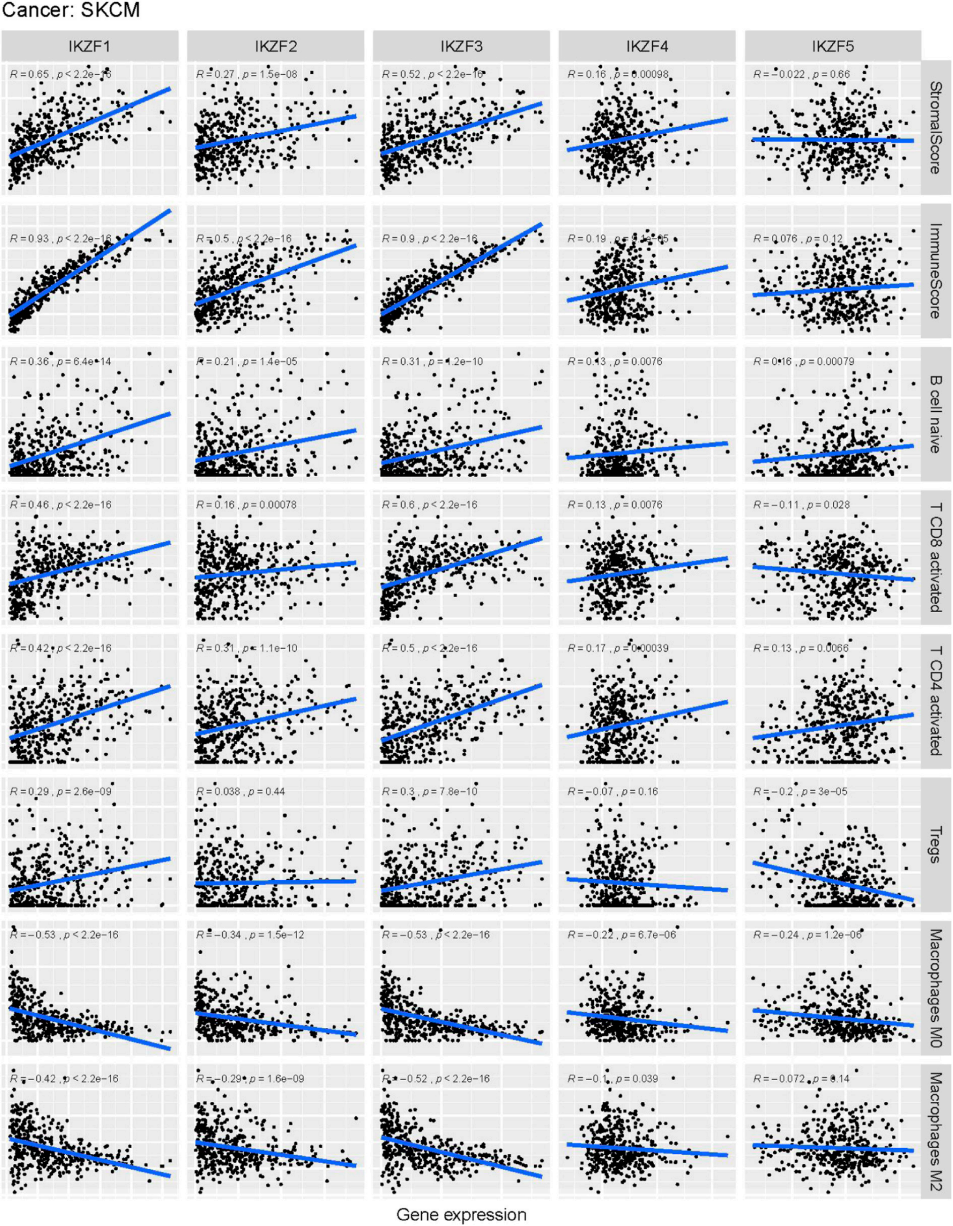
Correlation analysis of Ikaros family genes and the abundance of immune-infiltrating cells in the tumor microenvironment. Horizontal labels represent different levels of Ikaros gene expression, vertical labels represent different infiltrating immune cells, and the line represents the correlation between them. The correlation value is represented by the index R.

In the present study, we first determined the associations between patients’ overall characteristics and clinical prognosis with the expression of Ikaros across cancer types. We employed different analytical methods to identify the relationship between the transcriptional properties of *IKZF1–5* and different cancers (Figures 1–3). For patients with SKCM, the expression of *IKZF1–3* significantly decreased with increased T-stage or metastasis (Figure 4). Moreover, highly expressed *IKZF1–3*, but not *IKZF4–5*, were positively associated with a favorable prognostic outcome (Figure 5) of patients with SKCM through regulating immunocyte infiltration (Figure 9) and the immune response (Figure 6, 7). Together, these results indicate that highly expressed *IKZF1–3* gain clinical significance for predicting the prognosis of patients with SKCM, *IKZF3* is an independent prognostic factor of SKCM, thus suggesting that *IKZF3* are prognostic biomarkers and immunotherapeutic targets of melanoma. To the best of our knowledge, there is no prior study that comprehensively investigated the relationship between Ikaros gene expression and SKCM.

A limitation of the present study is the lack of experimental evidence that activation of *IKZF1–3*-related pathways predict the prognosis of SKCM. Therefore, detailed epigenetic regulation of Ikaros during the pathogenesis of melanoma should be further investigated.

In conclusion, our present study systematically describes the distributions and functions of Ikaros family genes across cancer types. Although the roles of other Ikaros family members such as *IKZF4–5* in melanoma remain unclear, *IKZF3* may serve as biomarkers for the outcomes of treatments for SKCM that achieve a positive clinical prognosis.

## Data Availability

The datasets presented in this study can be found in online repositories. The names of the repository/repositories and accession number(s) can be found in the article/Supplementary Material.

## Glossary

TME: Tumor microenvironment
TIL: Tumor-infiltrating lymphocyte
GSEA: Gene Set Enrichment Analysis
HR: Hazard ratio
OS: Overall survival
PFI: Progression-free interval
DSS: Disease-free survival
ACC: Adrenocortical carcinoma
BLCA: Bladder Urothelial Carcinoma
BRCA: Breast invasive carcinoma
CESC: Cervical squamous cell carcinoma and endocervical adenocarcinoma
CHOL: Cholangiocarcinoma
COAD: Colon adenocarcinoma
DLBC: Lymphoid Neoplasm Diffuse Large B-cell Lymphoma
ESCA: Esophageal carcinoma
GBM: Glioblastoma multiforme
HNSC: Head and Neck squamous cell carcinoma
KICH: Kidney Chromophobe
KIRC: Kidney renal clear cell carcinoma
KIRP: Kidney renal papillary cell carcinoma
LAML: Acute Myeloid Leukemia
LGG: Brain Lower Grade Glioma
LIHC: Liver hepatocellular carcinoma
LUAD: Lung adenocarcinoma
LUSC: Lung squamous cell carcinoma
MESO: Mesothelioma
OV: Ovarian serous cystadenocarcinoma
PAAD: Pancreatic adenocarcinoma
PCPG: Pheochromocytoma and Paraganglioma
PRAD: Prostate adenocarcinoma
READ: Rectum adenocarcinoma
SARC: Sarcoma
SKCM: Skin Cutaneous Melanoma
STAD: Stomach adenocarcinoma
TGCT: Testicular Germ Cell Tumors
THCA: Thyroid carcinoma
THYM: Thymoma
UCEC: Uterine Corpus Endometrial Carcinoma
UCS: Uterine Carcinosarcoma
UVM: Uveal Melanoma
MDS: Myelodysplastic syndrome
MM: Multiple myeloma
IMiD: Immunomodulatory drugs
CLL: Chronic lymphocytic leukemia

## References

[B1] Atkins M. B. Kunkel L. Sznol M. Rosenberg S. A. (2000). High-dose recombinant interleukin-2 therapy in patients with metastatic melanoma: Long-term survival update. Cancer J. Sci. Am. 6 (1), S11–S14. 10685652

[B2] Balch C. M. Buzaid A. C. Soong S. J. Atkins M. B. CasciNelliN. Coit D. G. (2001). Final version of the American Joint Committee on Cancer staging system for cutaneous melanoma. J. Clin. Oncol. 19 (16), 3635–3648. 10.1200/JCO.2001.19.16.3635 11504745

[B3] Besser M. J. Shapira-Frommer R. Treves A. J. Zippel D. Itzhaki O. Hershkovitz L. (2010). Clinical responses in a phase II study using adoptive transfer of short-term cultured tumor infiltration lymphocytes in metastatic melanoma patients. Clin. Cancer Res. 16 (9), 2646–2655. 10.1158/1078-0432.CCR-10-0041 20406835

[B4] Borgo C. D'Amore C. Sarno S. Salvi M. Ruzzene M. (2021). Protein kinase CK2: A potential therapeutic target for diverse human diseases. Signal Transduct. Target. Ther. 6 (1), 183. 10.1038/s41392-021-00567-7 33994545 PMC8126563

[B5] Chen J. C. Perez-Lorenzo R. Saenger Y. M. Drake C. G. Christiano A. M. (2018). IKZF1 enhances immune infiltrate recruitment in solid tumors and susceptibility to immunotherapy. Cell Syst. 7 (1), 92–103. 10.1016/j.cels.2018.05.020 29960886

[B6] Chen L. Niu Q. Huang Z. Yang B. Wu Y. Zhang J. (2020). IKZF1 polymorphisms are associated with susceptibility, cytokine levels, and clinical features in systemic lupus erythematosus. Med. Baltim. 99 (41), e22607. 10.1097/MD.0000000000022607 PMC7544280 33031316

[B7] Fan Y. Lu D. (2016). The Ikaros family of zinc-finger proteins. Acta Pharm. Sin. B 6 (6), 513–521. 10.1016/j.apsb.2016.06.002 27818917 PMC5071621

[B8] Ferlay J. Soerjomataram I. Dikshit R. Eser S. Mathers C. Rebelo M. (2015). Cancer incidence and mortality worldwide: Sources, methods and major patterns in GLOBOCAN 2012. Int. J. Cancer 136 (5), E359–E386. 10.1002/ijc.29210 25220842

[B9] Fu Q. Chen N. Ge C. Li R. Li Z. Zeng B. (2019). Prognostic value of tumor-infiltrating lymphocytes in melanoma: A systematic review and meta-analysis. Oncoimmunology 8 (7), 1593806. 10.1080/2162402X.2019.1593806 31143514 PMC6527267

[B10] Gao S. Wang S. Song Y. (2020). Novel immunomodulatory drugs and neo-substrates. Biomark. Res. 8, 2. 10.1186/s40364-020-0182-y 31938543 PMC6953231

[B11] He L. C. Gao F. H. Xu H. Z. Zhao S. Ma C. M. Li J. (2012). Ikaros inhibits proliferation and, through upregulation of Slug, increases metastatic ability of ovarian serous adenocarcinoma cells. Oncol. Rep. 28 (4), 1399–1405. 10.3892/or.2012.1946 22859015

[B12] Hodi F. S. O'Day S. J. McDermott D. F. Weber R. W. Sosman J. A. Haanen J. B. (2010). Improved survival with ipilimumab in patients with metastatic melanoma. N. Engl. J. Med. 363 (8), 711–723. 10.1056/NEJMoa1003466 20525992 PMC3549297

[B13] Hung J. J. Kao Y. S. Huang C. H. Hsu W. H. (2020). Author Correction: Overexpression of Aiolos promotes epithelial-mesenchymal transition and cancer stem cell-like properties in lung cancer cells. Sci. Rep. 10 (1), 1309. 10.1038/s41598-020-57957-0 31974483 PMC6978362

[B14] Iglesia M. D. Parker J. S. Hoadley K. A. Serody J. S. Perou C. M. Vincent B. G. (2016). Genomic analysis of immune cell infiltrates across 11 tumor types. J. Natl. Cancer Inst. 108 (11), djw144. 10.1093/jnci/djw144 27335052 PMC5241901

[B15] Javierre B. M. Rodriguez-Ubreva J. Al-Shahrour F. Corominas M. Grana O. Ciudad L. (2011). Long-range epigenetic silencing associates with deregulation of Ikaros targets in colorectal cancer cells. Mol. Cancer Res. 9 (8), 1139–1151. 10.1158/1541-7786.MCR-10-0515 21737484

[B16] John L. B. Ward A. C. (2011). The Ikaros gene family: Transcriptional regulators of hematopoiesis and immunity. Mol. Immunol. 48 (9-10), 1272–1278. 10.1016/j.molimm.2011.03.006 21477865

[B17] Lee N. Zakka L. R. Mihm M. C. Jr. Schatton T. (2016). Tumour-infiltrating lymphocytes in melanoma prognosis and cancer immunotherapy. Pathology 48 (2), 177–187. 10.1016/j.pathol.2015.12.006 27020390

[B18] Liu Y. Y. Ge C. Tian H. Jiang J. Y. Zhao F. Y. Li H. (2017). The transcription factor Ikaros inhibits cell proliferation by downregulating ANXA4 expression in hepatocellular carcinoma. Am. J. Cancer Res. 7 (6), 1285–1297. 28670491 PMC5489778

[B19] Locy H. de Mey S. de Mey W. De Ridder M. Thielemans K. Maenhout S. K. (2018). Immunomodulation of the tumor microenvironment: Turn foe into friend. Front. Immunol. 9, 2909. 10.3389/fimmu.2018.02909 30619273 PMC6297829

[B20] Megahed M. Schön M. Selimovic D. Schön M. P. (2002). Reliability of diagnosis of melanoma *in situ* . Lancet (London, Engl. 359 (9321), 1921–1922. 10.1016/S0140-6736(02)08741-X 12057558

[B21] Molnár A. Georgopoulos K. Molnar A. (1994). The Ikaros gene encodes a family of functionally diverse zinc finger DNA-binding proteins. Mol. Cell. Biol. 14 (12), 8292–8303. 10.1128/mcb.14.12.8292 7969165 PMC359368

[B22] Nazarian R. Shi H. Wang Q. Kong X. Koya R. C. Lee H. (2010). Melanomas acquire resistance to B-RAF(V600E) inhibition by RTK or N-RAS upregulation. Nature 468 (7326), 973–977. 10.1038/nature09626 21107323 PMC3143360

[B23] Newman A. M. Liu C. L. Green M. R. Gentles A. J. Feng W. Xu Y. (2015). Robust enumeration of cell subsets from tissue expression profiles. Nat. Methods 12 (5), 453–457. 10.1038/nmeth.3337 25822800 PMC4739640

[B24] Payne K. J. Dovat S. (2011). Ikaros and tumor suppression in acute lymphoblastic leukemia. Crit. Rev. Oncog. 16 (1-2), 3–12. 10.1615/critrevoncog.v16.i1-2.20 22150303 PMC3243972

[B25] Perdomo J. Holmes M. Chong B. Crossley M. (2000). Eos and pegasus, two members of the Ikaros family of proteins with distinct DNA binding activities. J. Biol. Chem. 275 (49), 38347–38354. 10.1074/jbc.M005457200 10978333

[B26] Pourabdollah M. Bahmanyar M. Atenafu E. G. Reece D. Hou J. Chang H. (2016). High IKZF1/3 protein expression is a favorable prognostic factor for survival of relapsed/refractory multiple myeloma patients treated with lenalidomide. J. Hematol. Oncol. 9 (1), 123. 10.1186/s13045-016-0354-2 27881177 PMC5120536

[B27] Rebollo A. Schmitt C. (2003). Ikaros, Aiolos and Helios: Transcription regulators and lymphoid malignancies. Immunol. Cell Biol. 81 (3), 171–175. 10.1046/j.1440-1711.2003.01159.x 12752680

[B28] Roma-Rodrigues C. Mendes R. Baptista P. V. Fernandes A. R. (2019). Targeting tumor microenvironment for cancer therapy. Int. J. Mol. Sci. 20 (4), E840. 10.3390/ijms20040840 PMC6413095 30781344

[B29] Siegel R. L. Miller K. D. Jemal A. (2016). Cancer statistics. Ca. Cancer J. Clin. 66 (1), 7–30. 10.3322/caac.21332 26742998

[B30] Soengas M. S. Lowe S. W. (2003). Apoptosis and melanoma chemoresistance. Oncogene 22 (20), 3138–3151. 10.1038/sj.onc.1206454 12789290

[B31] Sriaroon P. Chang Y. Ujhazi B. Csomos K. Joshi H. R. Zhou Q. (2019). Familial immune thrombocytopenia associated with a novel variant in IKZF1. Front. Pediatr. 7, 139. 10.3389/fped.2019.00139 31069201 PMC6491668

[B32] Sun D. Wang J. Han Y. Dong X. Ge J. Zheng R. (2021). Tisch: A comprehensive web resource enabling interactive single-cell transcriptome visualization of tumor microenvironment. Nucleic Acids Res. 49 (D1), D1420–d1430. 10.1093/nar/gkaa1020 33179754 PMC7778907

[B33] Sun L. Liu A. Georgopoulos K. (1996). Zinc finger-mediated protein interactions modulate Ikaros activity, a molecular control of lymphocyte development. EMBO J. 15 (19), 5358–5369. 10.1002/j.1460-2075.1996.tb00920.x 8895580 PMC452279

[B34] Tachita T. Kinoshita S. Ri M. Aoki S. Asano A. Kanamori T. (2020). Expression, mutation, and methylation of cereblon-pathway genes at pre- and post-lenalidomide treatment in multiple myeloma. Cancer Sci. 111 (4), 1333–1343. 10.1111/cas.14352 32061138 PMC7156787

[B35] Tsai K. K. Zarzoso I. Daud A. I. (2014). PD-1 and PD-L1 antibodies for melanoma. Hum. Vaccin. Immunother. 10 (11), 3111–3116. 10.4161/21645515.2014.983409 25625924 PMC4514131

[B36] Vitale C. Falchi L. Ten Hacken E. Gao H. Shaim H. Van Roosbroeck K. (2016). Ofatumumab and lenalidomide for patients with relapsed or refractory chronic lymphocytic leukemia: Correlation between responses and immune characteristics. Clin. Cancer Res. 22 (10), 2359–2367. 10.1158/1078-0432.CCR-15-2476 26733610 PMC5118034

[B37] Wang X. Xiong H. Liang D. Chen Z. Li X. Zhang K. (2020). The role of SRGN in the survival and immune infiltrates of skin cutaneous melanoma (SKCM) and SKCM-metastasis patients. BMC cancer 20 (1), 378. 10.1186/s12885-020-06849-7 32370744 PMC7201763

[B38] Weber J. (2010). Immune checkpoint proteins: A new therapeutic paradigm for cancer--preclinical background: CTLA-4 and PD-1 blockade. Semin. Oncol. 37 (5), 430–439. 10.1053/j.seminoncol.2010.09.005 21074057

[B39] Yang M. Liu Y. Mo B. Xue Y. Ye C. Jiang Y. (2019). Helios but not CD226, TIGIT and Foxp3 is a potential marker for CD4+ treg cells in patients with rheumatoid arthritis. Cell. Physiol. biochem. 52 (5), 1178–1192. 10.33594/000000080 30990587 PMC6943339

[B40] Yeoh A. Lu Y. Chin W. Chiew E. K. H. Lim E. H. Li Z. (2018). Intensifying treatment of childhood B-lymphoblastic leukemia with IKZF1 deletion reduces relapse and improves overall survival: Results of Malaysia-Singapore ALL 2010 study. J. Clin. Oncol. 36 (26), 2726–2735. 10.1200/JCO.2018.78.3050 30044693

[B41] Yoshida T. Georgopoulos K. (2014). Ikaros fingers on lymphocyte differentiation. Int. J. Hematol. 100 (3), 220–229. 10.1007/s12185-014-1644-5 25085254 PMC4492456

[B42] Yoshihara K. Shahmoradgoli M. Martínez E. Vegesna R. Kim H. Torres-Garcia W. (2013). Inferring tumour purity and stromal and immune cell admixture from expression data. Nat. Commun. 4, 2612. 10.1038/ncomms3612 24113773 PMC3826632

[B43] Zhao W. Chen T. B. Wang H. (2020). Ikaros is heterogeneously expressed in lung adenocarcinoma and is involved in its progression. J. Int. Med. Res. 48 (8), 300060520945860. 10.1177/0300060520945860 32787735 PMC7543118

[B44] Zhou H. Yan Y. Zhang X. Zhao T. Xu J. Han R. (2020). Ginseng polysaccharide inhibits MDA-MB-231 cell proliferation by activating the inflammatory response. Exp. Ther. Med. 20 (6), 229. 10.3892/etm.2020.9359 33149784 PMC7604739

[B45] Zhou J. Dudley M. E. Rosenberg S. A. Robbins P. F. (2005). Persistence of multiple tumor-specific T-cell clones is associated with complete tumor regression in a melanoma patient receiving adoptive cell transfer therapy. J. Immunother. 28 (1), 53–62. 10.1097/00002371-200501000-00007 15614045 PMC2175172

[B46] Zhou N. Gutierrez-Uzquiza A. Zheng X. Y. Chang R. Vogl D. T. Garfall A. L. (2019). RUNX proteins desensitize multiple myeloma to lenalidomide via protecting IKZFs from degradation. Leukemia 33 (8), 2006–2021. 10.1038/s41375-019-0403-2 30760870 PMC6687534

