# Supplementary figures and images for: Novel IKZF3 transcriptomic signature correlates with positive outcomes of skin cutaneous melanoma: A pan-cancer analysis
Source: Front Genet. 2022 Oct 24;13:1036402. doi: 10.3389/fgene.2022.1036402 (PMC9638148; doi:10.3389/fgene.2022.1036402)

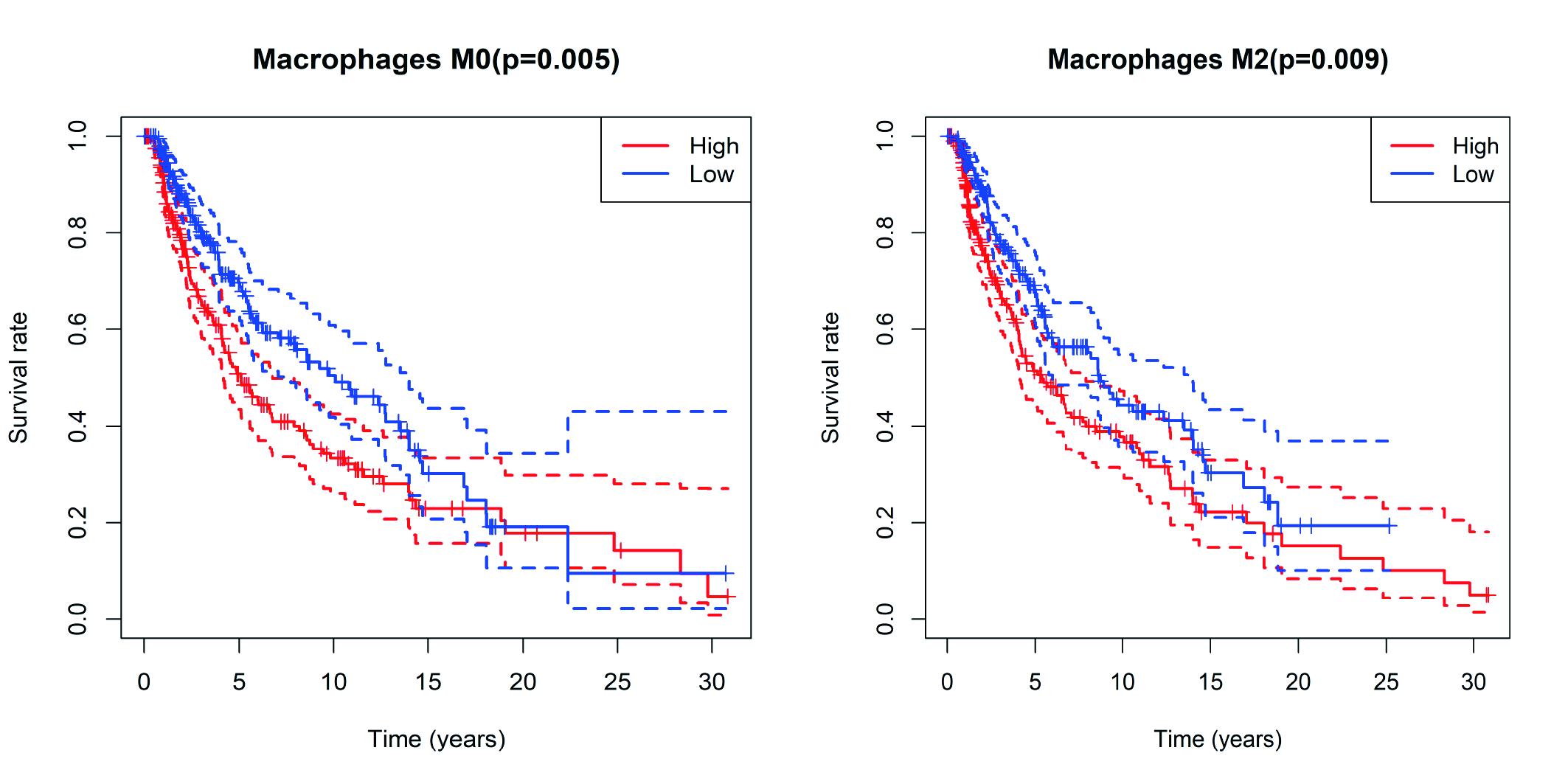

Supplement: Supplementary file 1 [file Image1.tif]

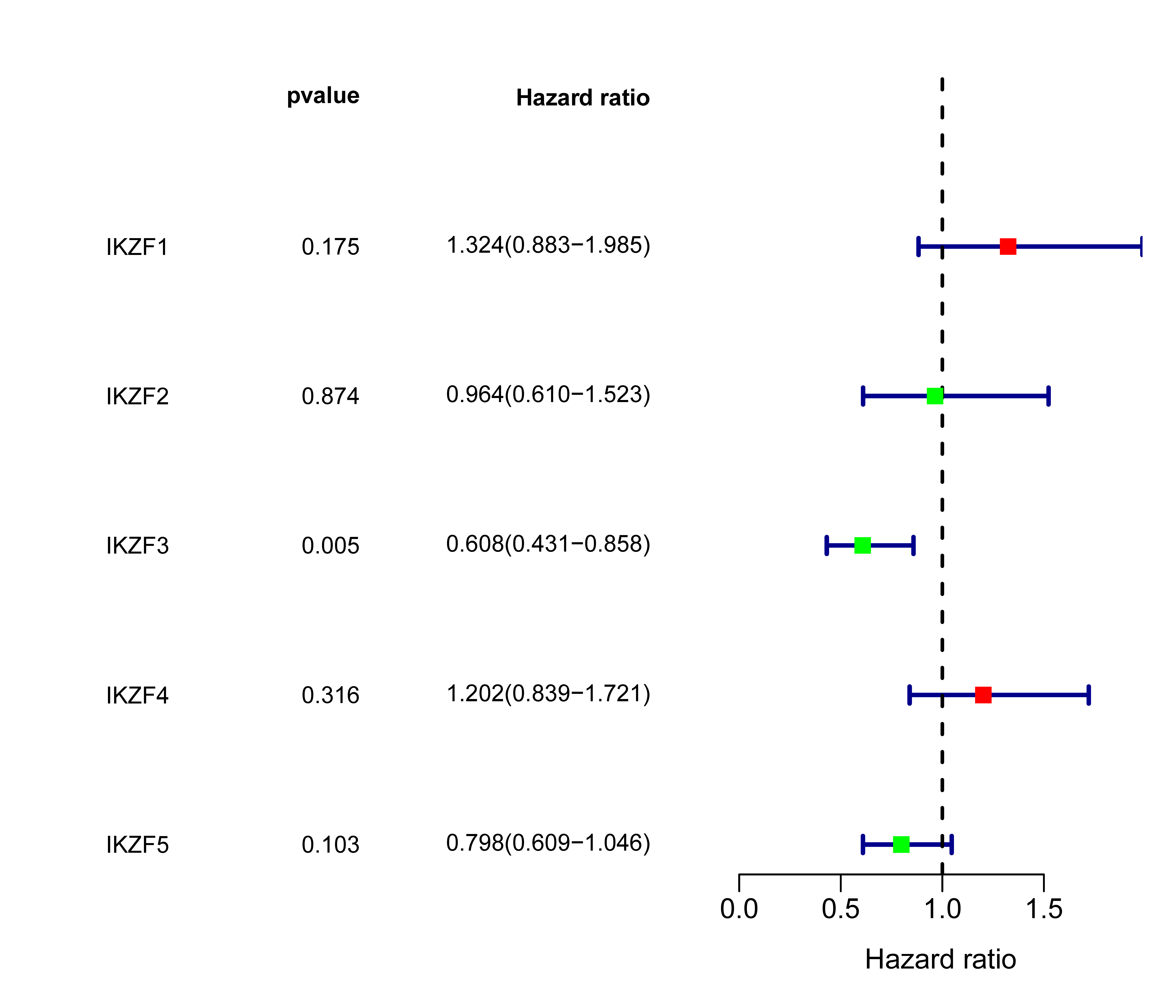

Supplement: Supplementary file 2 [file Image2.tiff]
